# Safety, efficacy, and operability of a newly developed absorbable adhesion barrier (GM142) in patients with primary rectal cancer scheduled for diverting ileostomy during laparoscopic surgery: Randomized controlled trial

**DOI:** 10.1002/ags3.12544

**Published:** 2022-01-05

**Authors:** Jun Watanabe, Shigeki Yamaguchi, Ichiro Takemasa, Masayoshi Yasui, Yasumitsu Hirano, Daisuke Nakano, Akio Shiomi, Shinya Munakata, Masanori Naito, Shunsuke Tsukamoto, Atsushi Ishibe, Yoshiaki Kuriu, Yasutake Uchima, Shinichiro Mori, Hideki Kanazawa, Go Wakabayashi, Takeshi Yamada, Muneaki Ezu, Masahiko Watanabe, Yusuke Kinugasa

**Affiliations:** ^1^ Department of Surgery Gastroenterological Center Yokohama City University Medical Center Yokohama Japan; ^2^ Department of Surgery Division of Colorectal Surgery Tokyo Women's Medical University Tokyo Japan; ^3^ Department of Surgery Surgical Oncology and Science Sapporo Medical University School of Medicine Hokkaido Japan; ^4^ Department of Gastroenterological Surgery Osaka International Cancer Institute Osaka Japan; ^5^ Department of Gastroenterological Surgery Saitama Medical University International Medical Center Saitama Japan; ^6^ Department of Surgery Tokyo Metropolitan Cancer and Infectious Diseases Center Komagome Hospital Tokyo Japan; ^7^ Division of Colon and Rectal Surgery Shizuoka Cancer Center Shizuoka Japan; ^8^ Department of Coloproctological Surgery Faculty of Medicine Juntendo University Tokyo Japan; ^9^ Department of Surgery Kitasato University Medical Center Saitama Japan; ^10^ Department of Gastroenterological Surgery St. Marianna University Yokohama West Hospital Yokohama Japan; ^11^ Department of Colorectal Surgery National Cancer Center Hospital Tokyo Japan; ^12^ Department of Gastroenterological Surgery Yokohama City University Graduate School of Medicine Yokohama Japan; ^13^ Department of Surgery Kyoto Prefectural University of Medicine Kyoto Japan; ^14^ Department of Surgery Fuchu Hospital Osaka Japan; ^15^ Department of Digestive Surgery, Breast and Thyroid Surgery Kagoshima University Graduate School of Medical Sciences Kagoshima Japan; ^16^ Department of Surgery Sagamihara National Hospital National Hospital Organization Kanagawa Japan; ^17^ Department of Surgery Ageo Central General Hospital Saitama Japan; ^18^ Department of Gastrointestinal and Hepato‐Biliary‐Pancreatic Surgery Nippon Medical School Tokyo Japan; ^19^ Medical Division Gunze Limited Tokyo Japan; ^20^ Department of Surgery Kitasato University Kitasato Institute Hospital Tokyo Japan; ^21^ Department of Gastrointestinal Surgery Tokyo Medical and Dental University Tokyo Japan

**Keywords:** antiadhesion barrier, GM142, hyaluronate‐carboxymethylcellulose, laparoscopic surgery, rectal cancer

## Abstract

**Aim:**

The aim of this study was to compare the outcomes of GM142, a newly developed gelatin film with a concave and convex structure to a commercially available conventional film, hyaluronate‐carboxymethylcellulose.

**Methods:**

Patients with primary rectal cancer who were scheduled for diverting ileostomy during laparoscopic surgery were eligible for this study. Patients were randomized before surgery and an antiadhesion film was applied under the umbilical incision. The primary outcome was the incidence of adhesion under the midline incision confirmed by second‐look surgery for diverting ileostomy closure. The secondary outcomes were the adhesion severity score, the extent of adhesion score, the presence of intestinal obstruction, and the success of all patching.

**Results:**

A total of 146 patients were enrolled. A total of 123 patients were included in the full analysis set. The primary outcome of “no adhesion” was observed in 66.1% in the GM142 group and 55.7% in the conventional film group. The noninferiority of GM142 to conventional film was confirmed (*P* = .0005). The secondary outcomes were similar between the groups. For the safety evaluation, there were no safety concerns regarding allergic reactions to gelatin or increased gelatin‐specific IgE antibody titers.

**Conclusions:**

The noninferiority of GM142 to conventional film was shown. GM142 showed no major safety issues. The clinical safety profiles of GM142 suggested certain physiological benefits of the gelatin film as an adhesion barrier.

## Graphical abstract

This randomized controlled trial revealed the noninferiority of a newly developed absorbable adhesion barrier (GM142) to conventional film. GM142 showed no major safety issues. The clinical safety profiles of GM142 suggested certain physiological benefits of the gelatin film as an adhesion barrier.

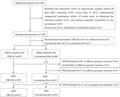

## 1 INTRODUCTION

Adhesion occurs frequently after abdominal operations, with an incidence rate of >90% in open surgery, and 35.6%–60% in laparoscopic surgery^1^, ^2^, ^3^ and is a common cause of bowel obstruction, chronic abdominal pain, and infertility.^4^ A systematic review estimated the incidence of postoperative adhesion in second‐look operations in patients undergoing abdominal surgery.^5^ The weighted mean rates of postoperative adhesion after gastrointestinal, gynecologic, and urologic surgery were estimated to be 66%, 51%, and 22%, respectively.

Several adhesion‐preventing absorbable barriers are currently available and a meta‐analysis investigation on the use of four adhesion prevention adjuvants (oxidized regenerated cellulose, hyaluronate‐carboxymethylcellulose, icodextrin liquid, and polyethylene glycol gels) in comparison to no treatment in abdominal surgery was conducted.^6^ Both oxidized regenerated cellulose and hyaluronate‐carboxymethylcellulose significantly reduced the incidence of site‐specific adhesion formation. Oxidized regenerated cellulose reduced the incidence of adhesion in gynecological surgery. The rate of serious adverse events (SAEs) was not investigated for any of the four agents.

GM142 is a newly developed thermally cross‐linked gelatin film with a concave and convex structure. The thermally cross‐linked gelatin film has improved the physical properties of the previously developed UV cross‐linked gelatin film^7^ and GM142 was designed to be suitable for laparoscopic surgery. Gelatin has excellent biocompatibility; however, previous chemical crosslinking methods were associated with toxicity caused by unreacted agents.^8^ Indeed, GM142 showed better physical strength and ductility, together with greater antiadhesive effects in comparison to the conventional hyaluronate‐carboxymethylcellulose film in animal models, without any cytotoxicity.^9^ The operability of GM142 in laparoscopic surgery was verified in a pig model and a test and repatch test showed the acceptability of repatching three times (results not published). A total of 117 GM142 gel sheets were used without any major safety concern. GM142 was easily inserted (even via a trocar), and easily placed and observed.

The aim of this prospective, randomized, multicenter, single‐blinded, parallel‐group, noninferiority study was to compare the newly developed GM142 to a commercially available conventional film, hyaluronate‐carboxymethylcellulose, in patients with primary rectal cancer scheduled for diverting ileostomy during laparoscopic surgery.

## 2 METHODS

### 2.1 Patients

This randomized controlled trial of GM142 was designed as a parallel‐group noninferiority study to compare GM142 and a conventional film in patients with primary rectal cancer who were scheduled for diverting ileostomy during laparoscopic surgery at 16 institutions in Japan. The study protocol was approved by the Ethics Advisory Committee and the Institutional Review Board of each participating hospital before its initiation. The study was registered in the Japanese UMIN Clinical Trials Registry as UMIN000034318 [http://www.umin.ac.jp/ctr/index.htm], and all patients provided written informed consent before registering in the study.

The inclusion criteria were as follows: (a) initial primary rectal cancer patient; (b) cStage 0–III; (c) scheduled for diverting ileostomy during laparoscopic rectal resection; (d) scheduled for closure of diverting ileostomy at 8‐48 wk after initial surgery; (e) age ≥20 y at the time of obtaining informed consent; and (f) signed informed consent. Among the 29 exclusion criteria described in the protocol, the following exclusion criteria were reported in this trial: No. 3 cStage IV; No. 16 use of unapproved medications or medical films within 12 wk prior to obtaining the informed consent; No. 24 patient regarded as unsuitable by the investigators; No. 26 no ileostomy; No. 27 specimens removed from site other than the midline incision; and No. 29 no application of GM142 or conventional film. The inclusion criteria and exclusion criteria are shown in Table S1.

Patient eligibility was checked using a web‐based registration system. The eligible patients were randomized and dynamically allocated to the GM142 group and the conventional film group at a 1:1 ratio using the minimization method (factors used for allocation: center, cStage, BMI, and presurgical chemotherapy).

### 2.2 Materials

**FIGURE 1:**
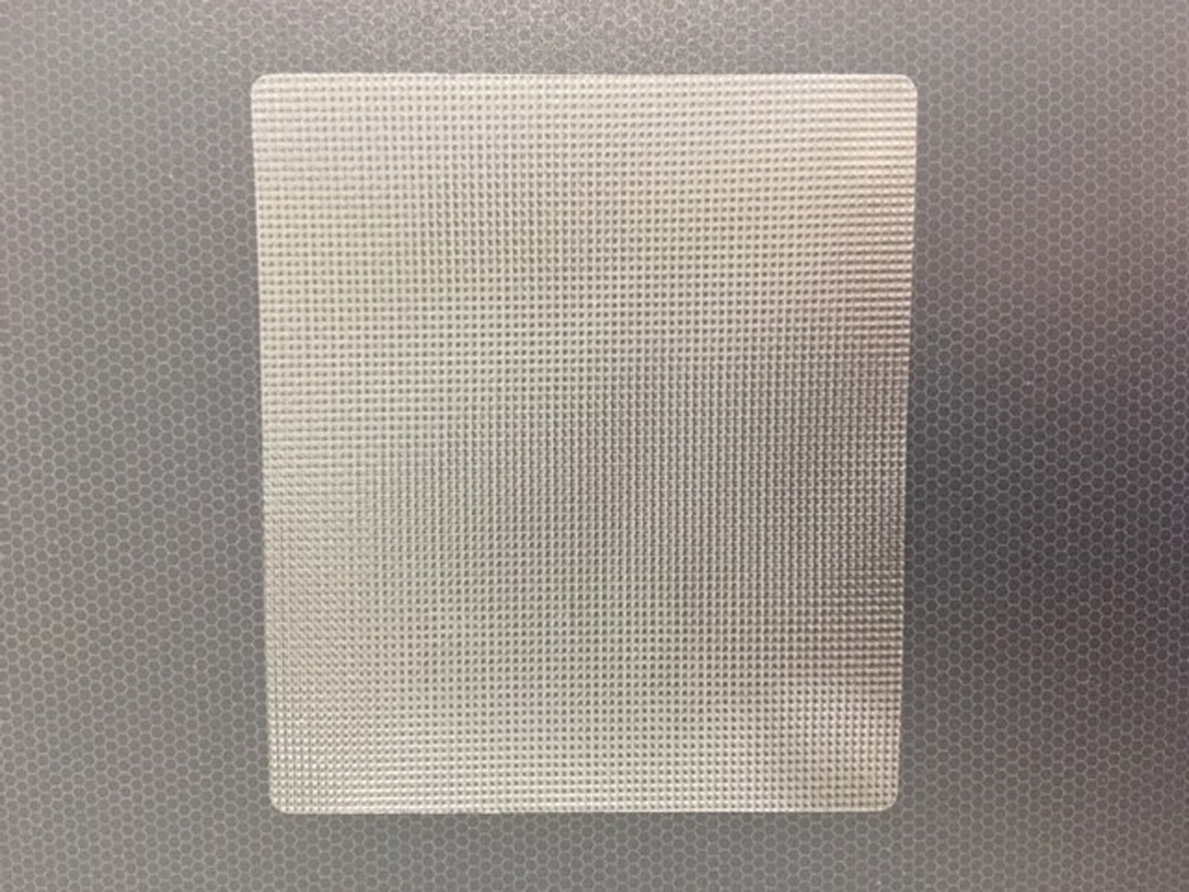
Surface‐textured gelatin film (GM142). GM142 is a bioabsorbable, surface‐textured, translucent, cross‐linked gelatin film prepared as 73.5 mm ×63.5 mm to be the same size as commercially available conventional film

GM142 is a bioabsorbable, surface‐textured, translucent, cross‐linked gelatin film prepared as 73.5 mm × 63.5 mm (Figure 1), to be the same size as commercially available conventional film. The conventional film is only one type of film. The product name is Seprafilm

### 2.3 Procedure

The film under investigation was used after abdominal surgery and applied just before closing the abdomen under the midline incision only. Wrapping to the site of the intestinal anastomosis was and should not be performed.

The patching method was as follows: (a) dry gloves or tweezers were used when handling the study film; (b) the film under investigation was inserted through the midline incision, minimizing contact at sites other than the application site and patched onto the tissue under the midline wound. The patching area was ~3 cm from both ends of the incision. If a single sheet was not sufficient to cover the entire target site, additional sheets were used. The maximum number of sheets was restricted to eight for both GM142 and conventional film in this trial.

### 2.4 Outcomes

The primary outcome was the incidence of adhesion under the midline incision and confirmed by second‐look surgery for diverting ileostomy closure. The adhesion‐related outcomes were judged by the independent professional committee using imaging data recorded during surgery.

The secondary outcomes were adhesion severity score (0: None, 1: Film‐like with no neovascularization, 2: Moderately thick with partial neovascularization, 3: Thick, solid adhesion with neovascularization), extent of adhesion score (0, none; 1, adhesion covers <50% of the target area/length; 2, adhesion covers >51% of the target area/length); and the presence of intestinal obstruction and success of all patching.

Adverse events (AEs) were coded and tabulated using MedDRA/J (v. 21.1) in system organ class and preferred term. The SAEs and causality of AEs were also tabulated. Laboratory test results (hematological tests, biochemical tests, and gelatin‐specific IgE antibody measures) were summarized by group with summary statistics, change from baseline, and abnormal changes.

Malfunctions included breakage of the sheet, rounding of the sheet, and any malfunction of the film, at any process of the trial such as delivery, storage, or during surgical operation. In an attempt to evaluate the operability of the antiadhesive sheet during the laparoscopic surgery, the following additional surgical data were evaluated: amount of bleeding, cut skin length, time for sheet patching, first surgical operation time, number of used sheets, number of successfully patched sheets at the target, adhesion around the temporary loop ileostomy, type of surgical operation (laparoscopic surgery, robot‐assisted surgery, with/without lateral lymph node dissection), region of ileostomy (upper or lower abdomen), target site of patched sheet (multiple choice; under the midline incision, around the diverting ileostomy, retroperitoneum, other), incidence rates of repatching, and sheet cutting.

### 2.5 Sample size calculation

The noninferiority margin was set to 18%, as approximately 1/4 of the effect size (72.8%) of conventional film reported for the Japanese Pharmaceuticals and Medical Devices Agency (PMDA) reexamination (2015). This noninferiority margin was also justified by the effect size of conventional film with laparoscopic surgery.^10^ Assuming the no‐adhesion rate (primary outcome) of both GM142 and conventional film was 86.7%, the noninferiority margin was 18%, with a two‐sided significance level of 5%, and 80% power; the sample size of each group was calculated as 56 patients. Assuming a discontinuation and withdrawal rate of 15%, a total of 132 patients were expected to be enrolled.

### 2.6 Statistical analysis

The difference of the no adhesion rate (primary outcome) between the GM142 group and the conventional film group was analyzed using a closed testing procedure (ie, noninferiority is confirmed when the lower limit of Wald‐type 95% confidence interval was ≥−18%, then superiority would be confirmed when the lower limit of Wald‐type 95% confidence interval is ≥0%). For the sensitivity analyses, a noninferiority test was performed using the Dunnett‐Gent method, the Clopper‐Pearson exact confidence interval was determined and Pearson's chi‐squared test was applied. For the secondary outcomes, malfunctions and other surgical data, categorical variables, and continuous variables were analyzed using Wilcoxon's signed rank test, and dichotomous variables were analyzed using Pearson's chi‐square test (two‐sided significance level: 5%). The full analysis set (FAS) was the primary analysis and the per‐protocol analysis set (PPS) was the secondary analysis. All statistical analyses were conducted using SAS v. 9.3 (SAS Institute, Cary, NC, USA).

## 3 RESULTS

### 3.1 Patient characteristics

**FIGURE 2:**
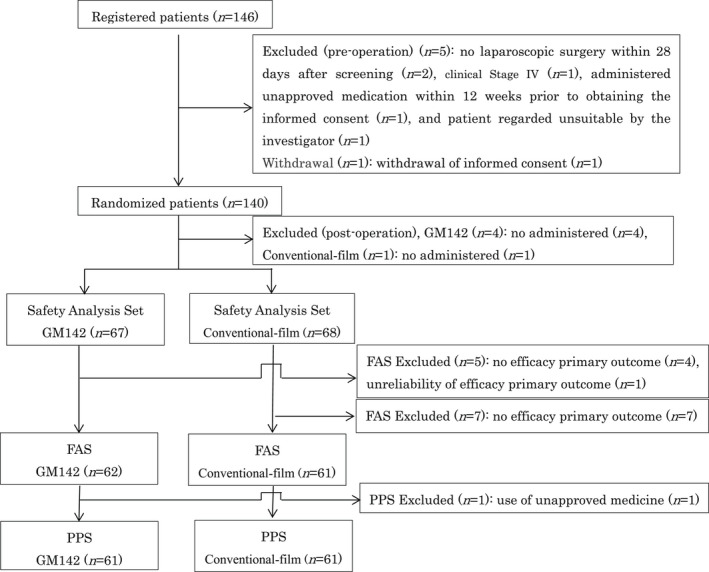
Patient flow chart. FAS, full analysis set; PPS, per protocol set

Between October 2018 and July 2020, a total of 146 primary rectal cancer patients scheduled for temporary loop ileostomy were enrolled. A total of six patients were excluded from randomization due to the following reasons: withdrawal of informed consent (n = 1), no laparoscopic surgery within 28 d after screening (n = 2), exclusion criterion No. 3 (n = 1), exclusion criterion No. 16 (n = 1), and exclusion criterion No. 24 (n = 1). A total of 140 patients were randomly allocated to the GM142 group or the conventional film group for laparoscopic surgery at a ratio of 1:1. A total of five patients were excluded after the operation. Consequently, 123 and 122 patients were included in the FAS and PPS, respectively (Figure 2).

There were no statistically significant differences between the GM142 and conventional film groups in sex, age, BMI, distance from anal margin, cStage, preoperative chemotherapy, history of appendectomy, hypertension, blood loss, or operative time, and the interval from first to second surgery. The median (1st quartile, 3rd quartile) of the total number of sheets used per patient in the GM142 and conventional film groups was 4 (4, 4) and 4 (4, 4) respectively (Table 1).

**TABLE 1:** Baseline characteristics (Safety Analysis Set)

	GM142 (n = 67)	Conventional‐film (n = 68)	*P*‐value
Gender, male/female	55 (82.1%)/12(17.9%)	61 (89.7%)/7(10.3%)	.203^†^
Age, years	63.0 (56.0, 73.0)	67.0 (56.3, 72.8)	.616^‡^
BMI, kg/m^2^	21.9 (20.6, 24.3)	22.9 (21.1, 25.1)	.158^‡^
<18.5	8 (11.9%)	7 (10.3%)	.339^‡^
18.5≦~<25	46 (68.7%)	43 (63.2%)	
25≦~<30	12 (17.9%)	15 (22.1%)	
30≦~	1 (1.5%)	3 (4.4%)	
Distance from anal margin, cm	6 (4, 8)	6 (5, 8)	.835^‡^
cStage, n (%)			.787^‡^
I	22 (32.8%)	23 (33.8%)	
II	22 (32.8%)	16 (23.5%)	
IIIa	9 (13.4%)	16 (23.5%)	
IIIb	14 (20.9%)	13 (19.1%)	
Preoperative chemotherapy	7 (10.4%)	9 (13.2%)	.616^†^
History of appendectomy	4 (6.0%)	4 (5.9%)	.983^†^
Hypertension	28 (41.8%)	33 (48.5%)	.432^†^
Diabetic mellitus	7 (10.4%)	15 (22.1%)	.068^†^
Blood loss, ml	12 (0, 66)	17 (5, 50)	.743^‡^
Operative time, min	284 (230, 400)	326 (244, 456)	.190^‡^
Total number of sheets used per patient	4 (4, 4)	4 (4, 4)	.452^‡^
Interval from first to second surgery, day^§^	122 (97, 178)	121 (91, 194)	.998^‡^

Note

Data are expressed as n (%) or median (1st quartile, 3rd quartile).

BMI, body mass index; FAS, full analysis set.

^†^Pearson's chi‐square test. ‡Wilcoxon signed rank test. §The first surgery is the operation of the laparoscopic rectal resection; the operation of the second surgery is the ileostomy closure with the clinical evaluation of antiadhesion effects.

### 3.2 Efficacy outcomes

**FIGURE 3:**
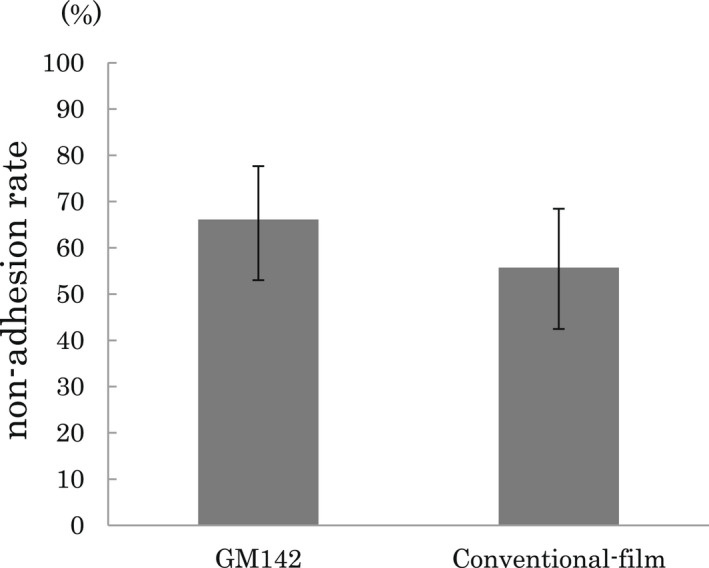
The efficacy primary outcomes of GM142 and conventional‐film (full analysis set). GM142 group; 66.1% (95% CI: 53.0%–77.7%) and conventional film group; 55.7% (95% CI: 42.4%–68.5%), the noninferiority of GM142 to conventional film (*P* =.0005, noninferiority margin: Δ = 0.18)

The primary outcome of no adhesion in the FAS was observed in 66.1% (95% confidence interval [CI]: 53.0%–77.7%) of the patients in the GM142 group and 55.7% (95% CI: 42.4%–68.5%) of the patients in the conventional film group (Figure 3). The results of the GM142 group were 10.4% better than the results of the conventional film group, and the noninferiority of GM142 to conventional film was confirmed (noninferiority margin: Δ = 0.18, *P* = .0005). However, this difference did not show superiority (*P* = .24). In the PPS, the noninferiority was also confirmed (*P* = .0003); however, the superiority (11.5%) of GM142 to conventional film did not show a statistical significance (*P* = .19).

The secondary outcomes were similar and did not differ to a statistically significant extent between the GM142 and conventional film groups (Table 2), eg, two intestinal obstructions in the GM142 group (3.2%) and three in the conventional film group (4.9%), 59 success of all patching in the GM142 group (95.2%) and 56 in the conventional film group (91.8%).

**TABLE 2:** Efficacy secondary outcomes (FAS)

	GM142 (n = 62)	Conventional‐film (n = 61)	*P*‐value
Adhesion severity score			
0: None	41 (66.1%)	34 (55.7%)	.315^†^
1: Film‐like with no neovascularization	5 (8.1%)	9 (14.8%)	
2: Moderately thick with partial neovascularization	12 (19.4%)	13 (21.3%)	
3: Thick, solid adhesion with neovascularization	4 (6.5%)	5 (8.2%)	
Extent of adhesion score			
0: None	41 (66.1%)	34 (55.7%)	.252^†^
1: Adhesions cover less than 50% of the target area/length	14 (22.6%)	18 (29.5%)	
2: Adhesions cover more than 51% of the target area/length	7 (11.3%)	9 (14.8%)	
Presence of intestinal obstruction	2 (3.2%)	3 (4.9%)	.635^‡^
Success of all patching	59 (95.2%)	56 (91.8%)	.450^‡^

Note

Data are expressed as n (%).

FAS, full analysis set.

^†^Wilcoxon signed rank test. ^‡^Pearson's chi‐square test.

### 3.3 Safety outcomes

A total of 186 AEs in 51 patients (76.1%) in the GM142 group were reported in comparison to 268 AEs in 62 patients (91.2%) in the conventional film group (Table 3). There was a significant difference between the GM142 and conventional film groups in the total number of reported AEs (Table 3). There were no statistically significant differences between the GM142 and conventional film groups in clinically important AEs such as anastomotic leakage, abdominal abscess, pelvic abscess, and wound infection. Regarding SAEs, nine events in nine patients (13.4%) in the GM142 group were observed in comparison to 29 events in 17 patients (25.0%) in the conventional film group (Table 3). In the conventional film group, all patients with SAEs recovered or were recovering/resolving, with the exception of one case of “mechanical obstruction”; the other SAEs were not related to the test films. A total of four AEs for which a causal relationship could not be denied were reported (Table 3), ie, three events in two patients (3.0%) in the GM142 group (paralytic ileus; paralytic ileus, abdominal abscess), and two events in two patients (2.9%) in the conventional film group (mechanical obstruction; paralytic ileus). Only “mechanical obstruction” in the conventional film group was severe and serious, whereas the others were nonserious.

**TABLE 3:** Summary of safety information

	GM142 (n = 67)	Conventional‐film (n = 68)	*P*‐value
Malfunction	10 (14.9%)	0 (.0%)	<.001^†^
Serious adverse event	9 (13.4%), 9 events	17 (25.0%), 29 events	.088^†^
Total number of adverse events	51 (76.1%), 186 events	62 (91.2%), 268 events	.018^†^
Clinically important adverse event			
Anastomotic leakage	3 (4.5%)	8 (11.8%)	.122^†^
Abdominal abscess	1 (1.5%)	0 (.0%)	.312^†^
Pelvic abscess	2 (3.0%)	2 (2.9%)	.988^†^
Wound infection	2 (3.0%)	2 (2.9%)	.988^†^
Test device related adverse event	2 (3.0%), 3 events	2 (2.9%), 2 events	.988^†^
Total number of abnormal changes in the biochemical tests at 5 d after surgery^‡^	6 (9.1%), 13 events	10 (14.9%), 22 events	.301^†^
The change from the baseline at 5 d after surgery (bleeding related hematological tests)			
Red blood cell count, 10^4^ μl	2 (−26, 23)	−21 (−42, 5)	.009^§^
Hemoglobin, g/dl	0.1 (−0.8, 0.8)	−0.6 (−1.4, 0.2)	.007^§^
Hematocrit, %	−0.3 (−3.1, 1.7)	−2.2 (−4.4, 0.2)	.012^§^

Note

Data are expressed as n (%), n (%) and events, or median (1st quartile, 3rd quartile).

FAS, full analysis set.

^†^Pearson's chi‐square test. ^‡^Evaluable cases, GM142: n = 66, Conventional‐film: n = 67. ^§^Wilcoxon signed rank test.

In the GM142 group, a total of 13 malfunctions were reported in 10 patients (14.9%) (Table 3), most of which occurred when the laminated aluminum package was opened. Among these malfunctions, 11 sheets were used without causing trouble, and two sheets were not used. These malfunctions did not cause any SAEs. No malfunction was reported in the conventional film group using commercially available sheets.

Abnormal changes in biochemical test results 5 d after surgery tended to be observed in fewer patients in the GM142 group (n = 6, 13 events) in comparison to the conventional film group (n = 10, 22 events) (Table 3). The majority of these abnormal changes were related to liver function (AST, ALT, γ‐GTP) and patients recovered or were recovering 4 wk after surgery. The change from baseline in bleeding‐related hematological test results (red blood cell count, hemoglobin, hematocrit) 5 d after surgery also suggested less disturbance in the GM142 group in comparison to the conventional film group (Table 3). These changes tended to continue 4 wk after surgery. It was considered that GM142 did not promote postoperative bleeding compared to the conventional film group. There were no safety concerns or abnormal changes in relation to gelatin‐specific IgE antibody, eg, change from baseline in the IgE antibody level 4 wk after surgery (Table 3).

## 4 DISCUSSION

The adhesion‐reducing effect of GM142 in comparison to conventional film in laparoscopic surgery was statistically confirmed in this randomized noninferiority study. The present study is the first report of a larger series to evaluate the adhesion‐reducing effect of GM142 in laparoscopic rectal surgery.

Gelatin is a denatured form of collagen, found in the connective tissue of both humans and animals.^9^ In rats, gelatin film showed a significantly higher antiadhesion effect without any cytotoxicity in comparison to the bioabsorbable cellulose‐based conventional film, which showed a certain degree of cytotoxicity depending on the in vitro test method.^9^, ^11^ Although the physiological/pathological adhesion process is very complicated, the inflammation process is expected to play an important role through fibrin and platelets.^12^ Due to the lysis of the film surface, both gelatin films and conventional films suppress fibroblasts proliferation. However, while conventional film extracts were cytotoxic, gelatin film extracts were not. It has been suggested that this difference may affect the antiadhesion effect.^9^

In clinical studies of the absorbable adhesion barriers, limited safety data have been reported, with most focusing on SAEs.^6^, ^13^ In general studies of bioabsorbable hemostats, regenerative collagen sheet/mesh showed good biocompatibility.^14^, ^15^, ^16^ In these studies, the gelatin‐based GM142 sheet was associated with fewer AEs than the conventional film, which was associated with less disturbance in laboratory test results. It is not clear whether these safety profiles are associated with better antiadhesion effects and/or with any postoperative morbidities, such as chronic pain or inflammatory complications.

Hyaluronate‐carboxymethylcellulose (conventional film), having low plasticity and tear easily, is difficult in repositioning and/or unrolling; thus, hyaluronate carboxymethylcellulose sheets are not suitable to be manipulated through trocars, as required in laparoscopic surgery.^13^, ^17^, ^18^ On the other hand, GM142 is designed for laparoscopic surgery. GM142 showed better physical strength and ductility than the conventional hyaluronic acid and carboxymethyl cellulose films in animal models.^9^ That is why carboxymethyl cellulose hyaluronate (conventional film) is not suitable from the viewpoint of operability in the case of trocar‐assisted laparoscopic surgery. GM142 is considered to have been proposed for laparoscopic surgery because of its excellent physical strength and ductility.

At present, reoperation/second‐look operations are commonly used for the clinical evaluation of antiadhesion effects, and it is difficult to conduct large and/or longer studies to investigate the relationship with adhesion related morbidities, such as small bowel obstruction, chronic pain, and female infertility. In an attempt to address these difficulties, the clinical adhesion score (CLAS) was recently developed.^19^ It will be interesting to follow up the findings suggested in this study in terms of the patients’ quality of life.^20^ Using CLAS, for example, the relationship with acute postoperative clinical signs/signals and chronic adhesion‐related morbidities may be discovered.

This noninferiority study was associated with several limitations; for example, trocars were not used, and for a fair comparison the size of the sheet was adjusted to that of the conventional film. The adhesion‐related efficacy evaluations were standardized by an independent professional committee; however, the surgical equipment, eg, robot‐assisted laparoscopic surgery and procedures varied considerably at each site, which made it difficult to evaluate the operability of GM142 and conventional‐film based on data collected during surgery. The observed malfunction of the GM142 trial package had been resolved in the commercial product.

In conclusion, the adhesion prevention effect of GM142 was confirmed to be noninferior to conventional film. GM142 showed no major safety issues and satisfactory operability during laparoscopic surgery. The clinical safety profiles of GM142 suggested certain physiological benefits of gelatin film as an adhesion barrier.

## DISCLOSURE

Funding: This study was funded by Gunze Limited, Chuo‐ku, Tokyo, Japan.

Conflicts of Interest: Drs. Jun Watanabe, Shigeki Yamaguchi, Ichiro Takemasa, Masayoshi Yasui, Yasumitsu Hirano, Daisuke Nakano, Akio Shiomi, Shinya Munakata, Masanori Naito, Shunsuke Tsukamoto, Atsushi Ishibe, Yoshiaki Kuriu, Yasutake Uchima, Shinichiro Mori, Hideki Kanazawa, Go Wakabayashi, Takeshi Yamada Masahiko Watanabe, and Yusuke Kinugasa have no conflicts of interest or financial ties to disclose. Muneaki Ezu is a member of the GUNZE LIMITED Medical Division R&D, Regulatory Affairs & Clinical Development from 2016 to date.

Approval of the research protocol: The study protocol was approved by the Ethics Advisory Committee and the Institutional Review Board of each participating hospital before its initiation.

Informed consent: All patients provided written informed consent before registering in the study.

Registry and the Registration No. of the study/Trial: The study was registered in the Japanese UMIN Clinical Trials Registry as UMIN000034318 [http://www.umin.ac.jp/ctr/index.htm].

Animal Studies: Not Applicable.

## Supporting information

Table S1
