# Supplementary material for: Safety, efficacy, and operability of a newly developed absorbable adhesion barrier (GM142) in patients with primary rectal cancer scheduled for diverting ileostomy during laparoscopic surgery: Randomized controlled trial
Source: Ann Gastroenterol Surg. 2022 Jan 5;6(4):515–22. doi: 10.1002/ags3.12544 (PMC9271016; doi:10.1002/ags3.12544)
Supplement: Supplementary file 1 — Table S1 [file AGS3-6-515-s001.docx]

**SUPPLEMENTAL TABLE 1** Inclusion Criteria and Exclusion Criteria

| **Inclusion Criteria**  1. Patients with initial and primary rectal cancer  2. Patients with cStage 0 to cStage III  3. Patients who are scheduled to undergo laparoscopic rectal resection in which a temporary ileostomy is performed.  4. Patients who are scheduled to undergo the closure of ileostomy 8 to 48 weeks after initial surgery.  5. Patients aged 20 years or older at the time of obtaining an informed consent  6. Patients who are fully informed about this study prior to the participation, understand the content, and give a written informed consent of their own free will  **Exclusion Criteria**  **(Preoperative criteria)**  1. Scheduled to undergo laparotomy for rectal resection.  2. Having a history of surgery in peritoneal cavity, except operation for appendicitis.  3. cStage IV  4. Scheduled to remove specimens from the site other than the midline incision.  5. Scheduled to undergo ileostomy at umbilical region.  6. ECOG performance status of 3 or greater  7. Peritonitis  8. Subject to emergency operation  9. Gelatin hypersensitivity  10. A history of severe drug allergy  11. Severe diabetes or anomaly of saccharometabolism  12. Significant obesity (BMI of 35 or higher)  13. Requiring chronic treatment with corticosteroids.  14. Scheduled to undergo chemotherapy within 2 weeks after initial surgery.  15. Scheduled to undergo surgery requiring hospital stay during the period from rectal resection to ileostomy closure.  16. Administered unapproved medications or medical devices within 12 weeks prior to obtaining the informed consent.  17. Pregnant or lactating, or wanting to become pregnant during clinical trials.  18. Undergoing dialysis due to hepatic cirrhosis, active hepatitis, or chronic renal failure.  19. Positive for any infection inappropriate for inclusion in the study based on the judgment of the principal investigator or sub-investigators.  20. Gastrointestinal perforations  21. Cancer with no schedule for radical surgery.  22. Psychiatric disease or symptom for which the enrollment in the study is judged unsuitable.  23. Considered less than one year of life expectancy.  24. Any other patients regarded unsuitable by the principal investigator or sub-investigators.  **(Perioperative / Discontinuance criteria)**  25. Having undergone laparotomy for rectal resection.  26. Having not undergone ileostomy.  27. Having removed specimens from the site other than the midline incision.  28. Having undergone ileostomy at umbilical region.  29. Having not applied either GM142 or Conventional-film. |
| --- |

Abbreviations: EOCG, Eastern Cooperative Oncology Group; BMI, Body mass index.
